# Influence of Zirconia and Organic Additives on Mechanical and Electrochemical Properties of Silica Sol-Gel Coatings

**DOI:** 10.3390/ma14092389

**Published:** 2021-05-04

**Authors:** Jolanta Gąsiorek, Anna Mazur-Nowacka, Anna Szczurek, Bartosz Babiarczuk, Wilhelm Jan Tic, Joanna Guziałowska-Tic, Jerzy Kaleta, Justyna Krzak

**Affiliations:** 1 Faculty of Mechanical Engineering, Wroclaw University of Science and Technology, Smoluchowskiego 25, 50-372 Wroclaw, Poland; anna.szczurek@pwr.edu.pl (A.S.); bartosz.babiarczuk@pwr.edu.pl (B.B.); jerzy.kaleta@pwr.edu.pl (J.K.); 2 Faculty of Chemistry, Wroclaw University of Science and Technology, Smoluchowskiego 25, 50-372 Wroclaw, Poland; anna.mazur-nowacka@pwr.edu.pl; 3 West Technology & Trading Polska, Sp. z o.o., Oświęcimska 100E, 45-641 Opole, Poland; Wilhelm.Tic@wttpolska.pl (W.J.T.); Joanna.Guzialowska-Tic@wttpolska.pl (J.G.-T.); 4 Department of Process and Environmental Engineering, Opole University of Technology, 45-758 Opole, Poland

**Keywords:** sol-gel, corrosion, SiO_2_, ZrO_2_, ORMOSIL

## Abstract

This paper presents the result of the investigation of organically modified silica (ORMOSIL)-zirconia coatings used to enhance their protective properties, namely corrosion and scratch resistance. Two different materials, i.e., SiO_2_/ZrO_2_ and SiO_2_/GPTMS/ZrO_2_, were synthesized, measured, and analyzed to find the difference in the used organosilane precursor (dimethyldiethoxysilane and (3-glycidoxypropyl)trimethoxysilane, respectively). SiO_2_/ZrO_2_ coatings showed higher hardness than SiO_2_/GPTMS/ZrO_2_. Moreover, the value of polarization resistance (R_p_) for SiO_2_/GPTMS/ZrO_2_ coated 316L steel relative to the uncoated one was obtained. It was nearly 84 times higher. The coating delamination was observed with load 16N. Additionally, the corrosion mitigation for 316L coated by SiO_2_/GPTMS/ZrO_2_ was observed even after extended exposure to corrosion agents.

## 1. Introduction

Many types of coatings, including inorganic or organic ones (paints, enamels, and lacquers) produced to protect against corrosion were developed as a result of modern technological advances. It should be added that the research that contributed to these latest scientific developments was conducted on corrosive degradation. The sol-gel method allows good corrosion protection capabilities to be attained thanks to the possibility of using the numerous types of sol-gel synthesis precursors, other reagents (e.g., inhibitors), and chemical or physical treatments (e.g., etching or polishing). It also makes it possible to obtain coatings with wear resistance properties in a corrosive environment [1,2,3,4,5,6,7]. More and more emphasis is being placed on developing fluoride-free materials for corrosion mitigation [5]. Taking into consideration the elimination of fluorinated compounds used in corrosion protection, the application of various sol-gel oxide matrices modified by organic groups (ORMOSIL), inhibitors [8,9,10], or nanoparticles [10,11,12] may be a successful alternative.

Inorganic sol-gel oxides are the simplest way to achieve corrosion mitigation [13]. This group of materials is characterized by good adhesion to metal substrates, thus they are often applied as interlayers [14]. During the research on such types of materials, it was discovered that they have the ability to create many Van der Waals interactions, which can be transformed into stable covalent bonds [15]. Additionally, inorganic coatings show better wear resistance than pure organic ones, e.g., those containing polymer compounds. Typical inorganic sol-gel coatings for corrosion mitigation are obtained as simple oxides, e.g., zirconium, titania, and silica [14,16]. Apart from SiO_2_ protective coatings [17,18], ZrO_2_-based coatings with self-healing properties deserve particular consideration [15]. Inorganic zirconia compounds also increase the long-term stability of coating materials [19], the resistance of wear [20,21], and, when deposited as interlayers in superconducting materials, they fulfill a particular role, i.e., they prevent electrical short circuits [22].

Nowadays, also hybrid organic–inorganic sol-gel coatings are very popular due to their high efficiency in protection against corrosion agents [17,23]. The organic part changes density and increases the flexibility of the oxide network, and the inorganic part is responsible for good adhesion to the substrate and for wear resistance (e.g., abrasion resistance enhancement), as stated in the previous paragraph.

Another advancement of sol-gel matrices is the possibility of incorporation, namely inhibitors, nanoparticles, or nanocontainers can be incorporated in the sol-gel layer’s matrix in order to increase or prolong the protective effect of sol-gel coatings [9,11]. Numerous authors discussed the protective properties of the organically modified silica (ORMOSIL) [24,25,26,27,28,29], which additionally shows their increased resistance to mechanical loads [8,30]. In addition to this, the ease of functionalization/modification, which may lead to obtaining self-healing properties [26,31,32,33] being the key to long-term protection, was demonstrated in the literature.

In this paper, the enhancement of the protection features obtained for a new surface modification of two types of steel—P265GH and 316L—were examined and discussed. The chemical composition of the obtained materials, i.e., organically modified silica-zirconia coatings, was tested using EDX and Raman spectroscopy. Surface morphology and homogeneity were analyzed using SEM. To characterize adhesion and mechanical properties, a scratch test and a nanoindentation test were applied. Corrosion resistance was tested by the linear polarization test at two different time points, i.e., at 0 h and 24 h test durations.

## 2. Materials and Methods

### 2.1. Preparation of Sol-Gel Materials

**Figure 1:**
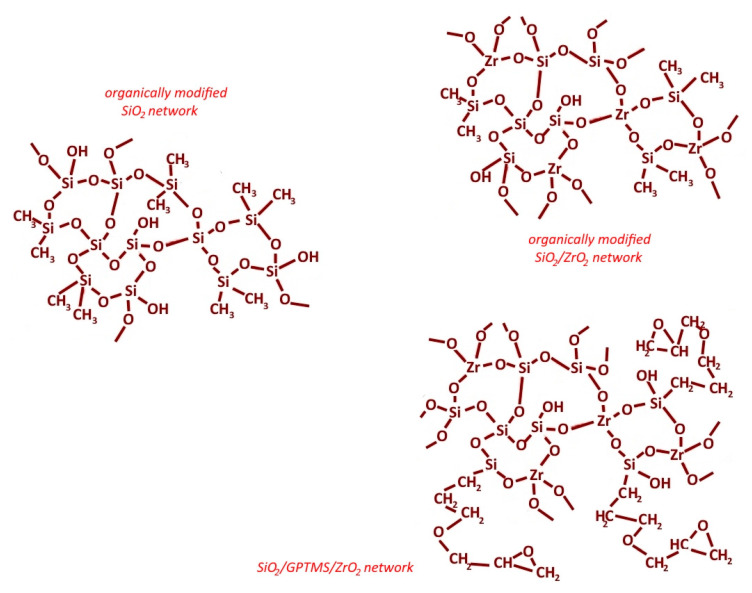
Block diagrams of obtained sol-gel oxide networks.

The silica and two different silica-zirconia hybrid materials were obtained by the sol-gel method with the reagents set as presented in Table 1. The organically modified SiO_2_ based matrices were prepared with tetraethoxysilane 98% (TEOS) and dimethyldiethoxysilane 98% (dMdEOS) as oxide precursors, both from Sigma Aldrich (Darmstadt, Germany). The base SiO_2_ sol was used as an interlayer and for further modification. The zirconia modification of organically modified silica matrices (SiO_2_/ZrO_2_) was obtained by mixing the base SiO_2_ (A) sol with ZrO_2_ (B) sol (after 1 h following reaction start, sol A was mixed with B, Table 1) based on zirconium(IV) butoxide 80% in *n*-butanol (ZrOBu, Sigma Aldrich). Both sols, SiO_2_ and ZrO_2_, were homogenized together for 0.5 h. Another kind of organically modified silica-zirconia hybrid (SiO_2_/GPTMS/ZrO_2_) was obtained by mixing an organically modified SiO_2_(C) sol with ZrO_2_ (B) sol (1 h after reaction start, C was mixed with B, Table 1), as mentioned above. The organically modified silica materials SiO_2_/GPTMS/ZrO_2_ were conducted by using (3-glycidoxypropyl)trimethoxysilane 97% (GPTMS, Alfa Aesar, Kandel, Germany) in a mixture with TEOS as a precursor. All materials were synthesized in acid condition with the use of chydrochloric acid 31–38% (HCl, Stanlab, Lublin, Poland) and/or acetic acid 99.5% (CH_3_COOH, Chempur, Piekary Śląskie, Poland) in amounts presented in Table 1. Additionally, to stabilize zirconia sol, by complexing a zirconium cation in the zirconium terakis-acetylacetone configuration [34] (it slows down hydrolysis and condensation), the acetyloacetone 99.6% (AcAc, Alfa Aesar) was added. The solvents for obtaining materials were ethanol 96% (EtOH, POCH, Gliwice, Poland) and butanol 99.9% (BuOH, POCH). All reagents were used as received without further purification. The used volume ratio and hydrolysates configuration are presented in Table 1 below. A block diagram of the obtained sol-gel network is presented in Figure 1.

### 2.2. Preparation of Steel Substrates

Structural, low carbon steel (P265GH) and austenitic, stainless steel (316L) were used as substrates. The chemical compositions of steels are shown in Table 2 (according to PN/EN 10027-2 and EN 10273). Before coating application, the surface of P265GH steel samples was sanded with sandpaper (grade 800) (R_a_ = 137 ± 14 nm after sanding, according to the PN-ISO 4288 standard with 20 measurement points per an individual sample, 0.8 mm length of measurement section, Olympus, LEXT OLS4000, Tokyo, Japan), cleaned in detergent and distilled water, and then etched in sulfuric acid (12%). After that, P265GH substrates were cleaned in distilled water and ethanol and left to dry at room temperature (20.5–21.5 °C). After sanding, the 316L steel samples (R_a_ = 194 ± 9 nm, according to the PN-ISO 4288 standard with 20 measurement points per an individual sample, 0.8 mm length of measurement section, Olympus, LEXT OLS4000) were rinsed in distilled water and etched in hydrochloric acid (9%), then rinsed with distilled water and ethanol and left to dry in room temperature (20.5–21.5 °C). The 316L steel surface was not sanded (assuming that coating would be deposited on an existing installation system in which an ideally treated surface was not possible).

### 2.3. Obtained Coatings and Stabilization

**Figure 2:**
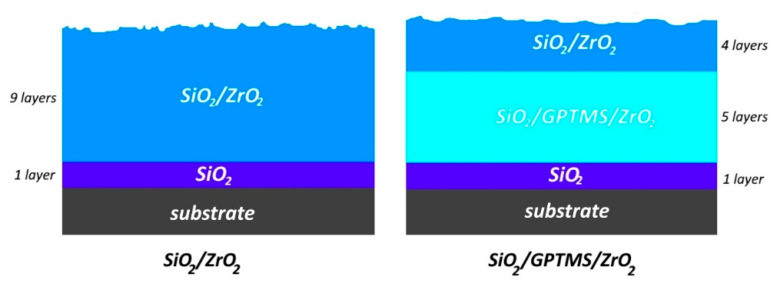
Scheme of layers deposition in the obtained coatings.

The ten-layer coatings were obtained by the dip-coating methods with controlled process parameters, such as dipping rate (34.12 mm/min), immersion time (60 s for the first layer, 30 s for the second layer, and 15 s for the other layers), and pulling out rate (34.12 mm/min). Each layer was deposited after a 24 h break at room temperature (20.5–21.5 °C) in open air. The sequence of layers in the obtained coatings is presented in Figure 2 (Figure 2 only represents how thick a given coating was during subsequent layers deposition). In both cases, the organically modified SiO_2_ interlayer (the layer nearest to the substrate) coatings was stabilized at 250 °C in open air (laboratory drying oven, POL-EOKO-APARATURA SP.J., SL 53 STD, Wodzisław Śląski, Poland). After the sixth and the tenth layers, the samples were dried as follows: SiO_2_/ZrO_2_ set was stabilized at 250 °C for 3 h, and SiO_2_/GPTMS/ZrO_2_ set was stabilized at 120 °C for 1 h in open air (POL-EOKO-APARATURA SP.J., SL 53 STD).

### 2.4. SEM and EDX

The surface morphology, the elemental composition, and the distribution of chemical elements of the obtained samples were examined by scanning electron microscopy (SEM) with an EDX detector (HITACHI S-3400N, Tokyo, Japan). For each sample, 3 measurement points were selected. The most representative results are presented in Section 3.1.

### 2.5. Raman Spectroscopy

The chemical composition and the types of bonding in the obtained materials were examined by Raman spectroscopy (Raman spectrometer LabRAM HR800 HORIBA JOBIN YVON, Lonqjumeau, France). The Raman spectra were measured in the range 100–4000 cm^−1^ using an argon laser with a 514.5 nm wavelength. For the individual samples, 6 measured points were performed. The most representative spectra are presented in the manuscript.

### 2.6. Scratch Test

The adhesion of the obtained coatings was determined with Micro Combi Tester (MCT), CSM Instruments, Graz, Austria according to the PN-EN 1071-3:2007 standard. A Rockwell indenter, Graz, Austria with a diameter of 100 μm was used to perform 3 scratches on individual samples, and each of them was 3 mm long. A load in the range from 30 mN to 20 N with linearly increasing speed of 1.5 mm/min was used. During the measurement, normal force (F_n_), friction coefficient (µ), and penetration depth (Pd) were recorded. Based on these parameters (FN, µ, Pd), the adhesion of the coatings to the substrate was specified, with characteristic loads (LC): LC_1_—cracking of coating, LC_2_—characteristic chipping of coatings, LC_3_—penetration of the coating into the substrate in the middle of the scratch.

### 2.7. Nanointendation Test

Hardness and the Young modulus were specified by the nanoindentation test, using Micro Combi Tester (MCT), CSM Instrument, with a pyramidal Berkovich tip. ISO 14577-1 standard was used to perform nanoindentation tests. The tests were taken by the load of the normal force of 0.1 mN and the rate of loading of 0.6 mN/min. For an individual sample, 12 measure points with a 15 s pause between loading and unloading were performed. The Young modulus was specified using the Oliver–Pharr method.

### 2.8. Electrochemical Test

Electrochemical tests were carried out in 3% NaCl solution after 0 h and 24 h of sample exposure in the solution with an SI 1286 potentiostat, Schlumberger, Houston, TX, USA. A three-electrode system with a calomel electrode as the reference electrode and a platinum electrode as the auxiliary electrode was used. The stationary potential and subsequently the polarization resistance, which used the Stern–Geary law (defined by R_p_ = ΔE/Δi equation) in the range of −0.015 V to +0.015 V, were measured.

The open circuit potential (OCP) was recorded until changes were smaller than 5 mV/5 min. The polarization curves were determined in the potential range from −0.75 V to +0.5 V relative to the saturated calomel electrode (SCE). The investigated samples were polarized at a potential change rate of 1 mV/s.

## 3. Results and Discussion

### 3.1. SEM and EDX

**Figure 3:**
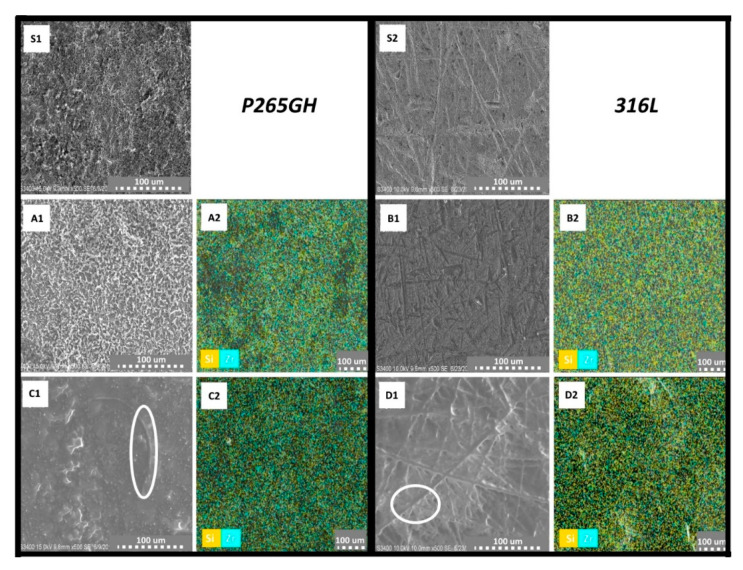
SEM images and EDX mapping for uncoated P265Gh steel (**S1**) and uncoated 316L steel (**S2**) SiO_2_/ZrO_2_ coatings (**A1**,**A2**) on P265GH steel and (**B1**,**B2**) on 316L steel, SiO_2_/GPTMS/ZrO_2_ coatings (**C1**,**C2**) on P265GH steel and (**D1**,**D2**) on 316L steel.

Figure 3 shows the morphology of uncoated P265GH (S1) and 316L(S2) steels. The surface of P265GH steel was characterized by the significant growth of surface area and porosity. Characteristic features were observed on the uncoated 316L surface. The possible reason for the occurrence of these features is described in Section 2.2. Figure 3 shows the morphology of the obtained coatings on both steel substrates. The sample with a SiO_2_/ZrO_2_ coating (Figure 3A1) was characterized by a significant growth of structure. This meant that the specific surface area increased, which may have increased the number of potential points of interaction between the corrosive medium and the sample surface [35]. The sample with a SiO_2_/GPTMS/ZrO_2_ coating (Figure 3C1) was smoother with some inhomogeneous inclusions (marked by white circles). These inclusions were not located on the coating surface (SiO_2_/ZrO_2_) but in the volume of the coating (SiO_2_/GPTMS/ZrO_2_). Both coatings, SiO_2_/ZrO_2_ (Figure 3A2) and SiO_2_/GPTMS/ZrO_2_ (Figure 3C2), on P265GH substrates were characterized by a regular distribution of Si and Zr, as can be seen in the result of EDX mapping. For both coatings on 316L substrates (Figure 3B1,D1), the inclusions were not observed. Furthermore, the result of EDX mapping (Figure 3B2,D2) also showed the regular distribution of Si and Zr in SiO_2_/GPTMS/ZrO_2_, similarly as in the case of SiO_2_/ZrO_2_ (Figure 3A2,C2).

### 3.2. Raman Spectroscopy

**Figure 4:**
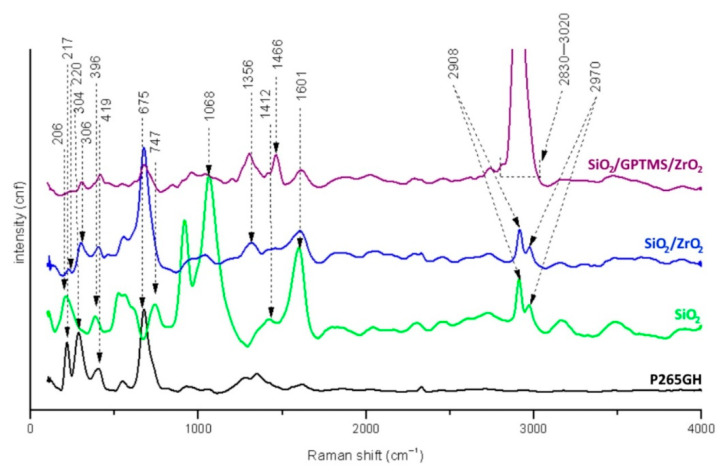
Raman spectra for P265GH steel uncoated and with sol-gel coatings.

Figure 4 shows the Raman spectra for uncoated carbon steel P265GH and the steel with coatings. For the uncoated steel samples, the characteristic bands for oxide iron were detected.

The strong band at 217 cm^−1^ and the medium band at 1356 cm^−1^ for γ-FeOOH (lepidocrocite), at 304 and 419 cm^−1^ for α-Fe_2_O_3_ (hematite), and at 675 cm^−1^ for Fe_3_O_4_ (magnetite) were detected [36,37]. The characteristic bands for silica sol-gel matrices were detected for the SiO_2_ coating at 206 cm^−1^ for Si–O–Si [38] and at 396 cm^−1^ for Si–O–Si from multimembered rings of oligosiloxanes [38,39,40]. The asymmetric vibrations of Si–O–Si were detected at 1068 cm^−1^ for all coatings [41]. Additionally, the used precursors contained a non-hydrolyzable part CH_3_ (non-hydrolyzable methyl groups from dimethyldiethoxysilane molecule) which was detected at 747 cm^−1^ and at 1412 cm^−1^ [38,42]. The vibrations of the methylene group –CH_2_ detected at 1466 cm^−1^ came from long aliphatic hydrocarbons of the broken epoxy ring (from GPTMS) [38,40,41,42]. These results indicated that part of the epoxy rings from GPTMS may have broken during the heat treatment [43]. Additionally, the large increase in the intensity of bands in the range of 2830–3020 cm^−1^ was observed, which may have meant that, besides the –OH groups for which this region is characteristic, the C–H vibrations of unreacted epoxy rings from GPTMS were also detected [44]. At 2908 and 2970 cm^−1,^ bands for symmetric and asymmetric vibrations, respectively, of CH_3_– methyl groups bonded to the silica network were detected [38,41]. For SiO_2_/ZrO_2_ spectra, weak signals at 220 cm^−1^ from tetragonal zirconia (t-ZrO_2_) [45] and 306 cm^−1^ from monoclinic zirconia (m-ZrO_2_) were observed [45,46,47,48]. For all spectra, the shift near 1601 cm^−1^ was observed. It seemed to represent the vibration interactions of OH from the absorbed water molecules [49].

**Figure 5:**
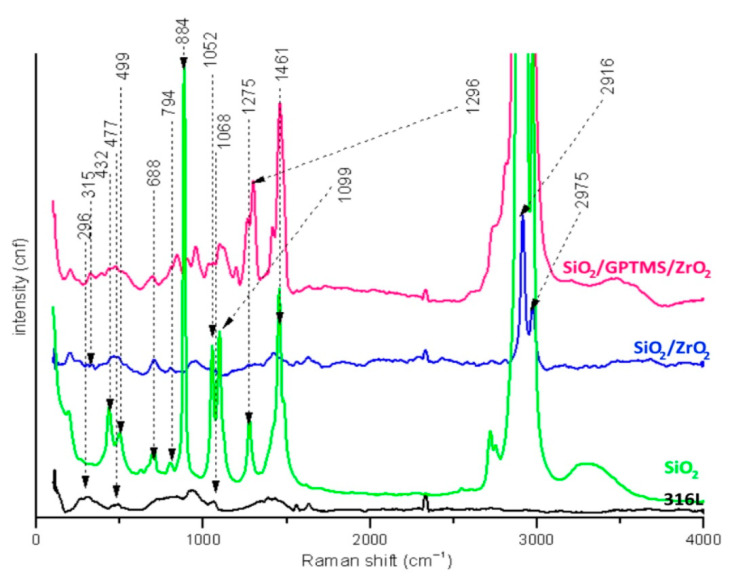
Raman spectra for 316L steel uncoated and with sol-gel coatings.

Figure 5 shows the Raman spectra of the uncoated 316L steel and the steel with sol-gel coatings. For the 316L spectrum, the characteristic signals were present at 296 cm^−1^ from α-Fe_2_O_3_ [50], at 477 and 1068 cm^−1^ from α-FeOOH, or at 477 cm^−1^ from Fe[CrO_4_]_3_ [36,51]. The signals at 499 cm^−1^ from the Si–O–Si [40], at 794 cm^−1^ from Si–O–Si [38], at 688 cm^−1^ from hydrolyzed precursor moieties ((CH_3_)_2_Si(OC_2_H_5_)_2_) [40], and at 1275 cm^−1^ of the deformation vibration of CH_3_ from the non-hydrolyzed part were detected [45]. Bands at 1296 cm^−1^ and 1461 cm^−1^ may have resulted from CH_3_ and CH_2_ vibrations. However, it is also possible that they were connected with the non- hydrolyzed part of precursors or they originated from the fragmentary broken epoxy ring [38,41,44]. Ethanol molecules had characteristic signals at 432 cm^−1^ and 884 cm^−1^ and double bands at 1052 cm^−1^ and 1099 cm^−1^ [40,41]. The strong band at 2900 cm^−1^ came from –OH from sol-gel matrices, non-reacted alcohol, or CH_3_ bond vibrations from the silica network [38,40,42]. For both spectra, SiO_2_/ZrO_2_ and SiO_2_/GPTMS/ZrO_2_ appeared at 315 cm^−1^, and then weak signals were observed; this possibly resulted from t-ZrO_2_ vibration [48,52].

### 3.3. Scratch Test

**Figure 6:**
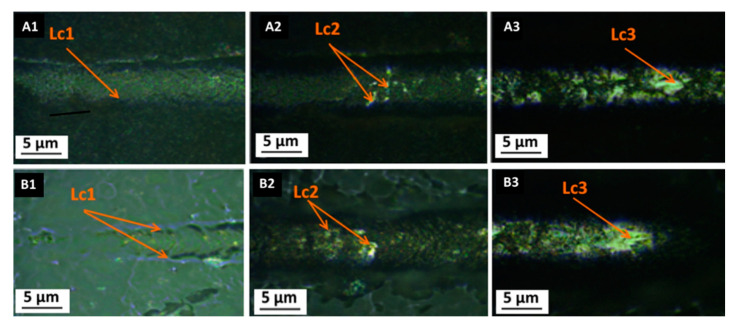
Images after scratch-test for P265GH steel with: (**A1**–**A3**) SiO_2_/ZrO_2_ coating, (**B1**–**B3**) SiO_2_/GPTMS/ZrO_2_ coating (L_C1_—characteristic crack of coating, L_C2_—chipping of coatings, L_C3_—penetration of the coating into the substrate in the middle of the scratch).

**Figure 7:**
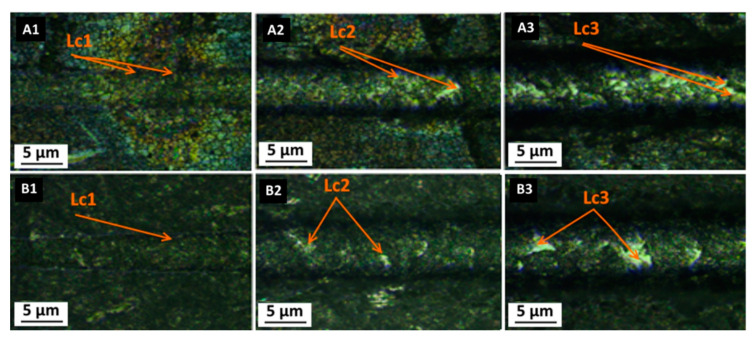
Images after scratch-test for 316L steel with: (**A1**–**A3**) SiO_2_/ZrO_2_ coating, (**B1**–**B3**) SiO_2_/GPTMS/ZrO_2_ coating (L_C1_—characteristic crack of coating, L_C2_—characteristic chipping of coatings, L_C3_—penetration of the coating into the substrate in the middle of the scratch).

The scratch resistance and the damages resulting from the tests are shown in Figure 6 and Figure 7. The coatings with organic compounds (SiO_2_/GPTMS/ZrO_2_) showed elastic deformations along the cracks, which was consistent with the literature data [17]. Additionally, for this type of coating, acoustic emission was not recorded during the test, which indicated the lack of brittle cracking of the coating materials [17]. For inorganic coating materials (SiO_2_/ZrO_2_), the deformations characteristic for the fragile type of destruction were observed. In particular, the acoustic emission and the deformed cracks of the conformal type were detected. Both of the obtained materials showed similar adhesion to both types of steel, i.e., P265GH and 316L. Each of the analyzed deformation characteristics for the coatings on P265GH steel were also observed for the coatings on the 316L steel. This is a valuable result, as it is extremely rare in the literature and indicates the wide applicability of the used coatings. The value of critical forces (L_c1_, L_c2_, L_c3_) for the specific damages of coatings on P265GH corresponded with the results obtained for the 316L coated samples. The first cracks (L_c1_) on both types of coatings were recorded under the load of 1.4 N (Table 3, Figure 6 and Figure 7). The highest value of the loading force at which coating delamination was observed was recorded for SiO_2_/GPTMS/ZrO_2_ (Figure 6B3, Figure 7B3). The delamination of this coating occurred with the load force value twice as high as SiO_2_/ZrO_2_. These results demonstrate that the first cracks for the analyzed coatings were detected at the similar load force value, however, their specifications were different:

- for SiO_2_/ZrO_2_, the characteristic coating cracks were observed at the edges of scratches; they seemed to conform to the groove, which meant conformal cracking (PN-EN 1071-3:2007).
- for SiO_2_/GPTMS/ZrO_2_, the cracks were observed within the groove, which indicated Hertz cracking (PN-EN 1071-3:2007).

Interestingly, the obtained sol-gel SiO_2_/GPTMS/ZrO_2_ coatings showed adhesion similar to gradient-oxide coatings obtained by laser radiation—the layer in the focal plane (“yellow_d” and “orange_d”) [53]. It should be noted that the mechanisms of the synthesis of sol-gel coatings and laser-oxidized coatings were completely different. The laser-oxidized coatings resulted from the oxidation of the substrate surface, which meant strong physicochemical interaction between the obtained coating and the substrate. In addition, this mechanism made the obtained materials very adhesive [53]. The oxide coatings originating from the sol-gel synthesis were produced by different mechanisms, which resulted in the predominance of electrostatic and weak hydrogen interactions in the first step (and covalent ones after heat treatment) at the coating-substrate level [15]. Thus, the sol-gel method allows one to obtain coatings characterized by high adhesiveness to metal substrates.

### 3.4. Nanointendation Test

**Figure 8:**
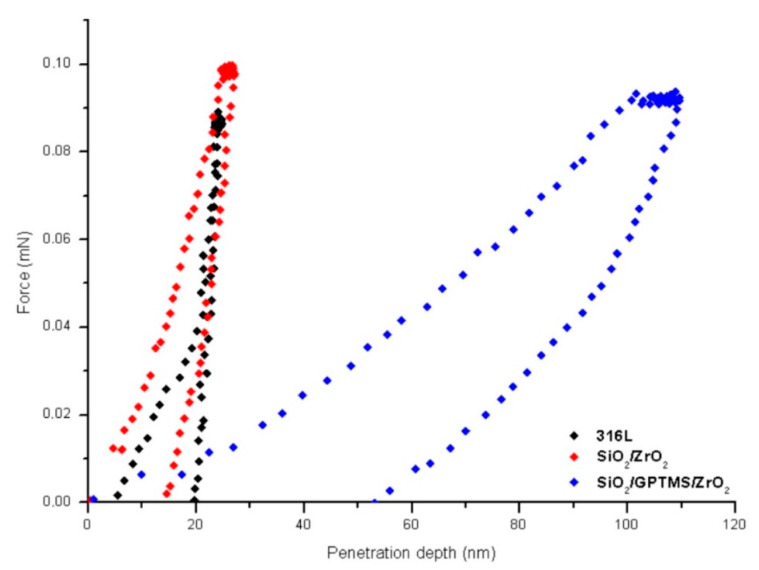
The load-displacement curves for of 316L steel with SiO_2_/GPTMS coatings.

The Young modulus (E_IT_) and hardness (H_IT_) were calculated for the maximum load of 0.1 mN with the Oliver–Pharr protocol; the results are presented in Table 4. The loading and the unloading curves for the 316L steel with coatings are shown in Figure 8.

The analysis of the obtained results indicates that the mechanical properties of SiO_2_/ZrO_2_ coating are similar to the surface of the uncoated 316L steel. The data obtained for the coating based on zirconia showed that its penetration depth was about six times lower (with the same force), which meant that the hardness of this coating was also significantly higher than in the case of SiO_2_/GPTMS/ZrO_2_. For the coating with zirconia, the mean Vickers hardness was about 236 ± 14 HV for 316L and about 236 ± 22 HV and 41 ± 12 HVSiO_2_/GPTMS/ZrO_2_ (according to the Oliver–Pharr procedure [54]). It was expected that the obtained E and H would result from the structure of sol-gel materials as, in general, the SiO_2_/ZrO_2_ consist of an inorganic network with CH_3_ moieties, and here, the inorganic part is dominant. Elasticity depends more on the organic part of the network. In the case of hardness, the organic part gains the advantage of being more influential. Both statements are supported by the results obtained for the SiO_2_/GPTMS/ZrO_2_ coating, where, during the growth of the organic part of networks, it was observed that the value of hardness decreased. In other words, the behavior observed for coating materials in the plastic and elastic region differed significantly from the behavior of the substrate without a coating. To sum up, in the case of oxide thin films on metallic substrates, H and E were influenced by the amount of organic part moieties in the network structure [55]. It may also be concluded that the crystal phase increased in the inorganic part of the sol-gel, observed in particular for SiO_2_/ZrO_2_, which had impact on the H value. The results showed that the addition of zirconia led to an increase in hardness [56]. The course of the nanoindentation curves (Figure 8) indicated that all samples had elastic-plastic features.

### 3.5. Electrochemical Test

The following results were obtained from the measurements in 3% NaCl: open circuit potential, polarization resistance (Rp), and polarization curves (Tafel plots). The density corrosion current was calculated based on the polarization resistance measurement using the Equation (1):

(1) $$I_{\mathrm{corr}}=\frac{\mathrm{ba}\times \mathrm{bc}}{2.3\left(\left|\mathrm{ba}\right|+\left|\mathrm{bc}\right|\right)}\times \frac{1}{\mathrm{Rp}}=\frac{B}{\mathrm{Rp}}$$

where:

R_p_—resistance polarization,

I_corr_—corrosion current densities,

b_c_/b_a_—cathodic and anodic Tafel’s parameters (established that b_c_/b_a_ *=* 0.120 V/dec).

The polarization resistance measurements were also used to E_corr_. Figures 11B, 12B, 15B and 16B show polarization curves—Tafel plots. However, due to the fact that Tafel’s method was used to calculate the I_corr_ for pure metals and that the 316L steel was characterized by complex composition (additions such as Mo, Ni, Cr), which generated errors during the calculation of the characteristic R_p_, I_corr,_ and the determination of E_corr_, these values were calculated using polarization resistance measurements. In addition, the Tafel plots could be distorted by concentration polarization, resistance drop effects, passivation phenomena, and complex redox reactions observed on the alloy steel surface. However, the Tafel plots also presented results such as polarization resistance in the widest range of potential.

**Figure 9:**
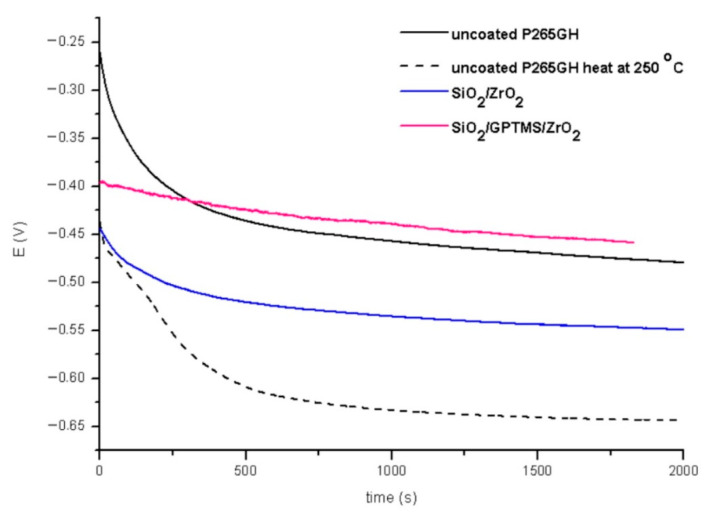
Open circuit potential for P265GH steel without coating and with oxide layers after duration in 3% NaCl 0 h.

**Figure 10:**
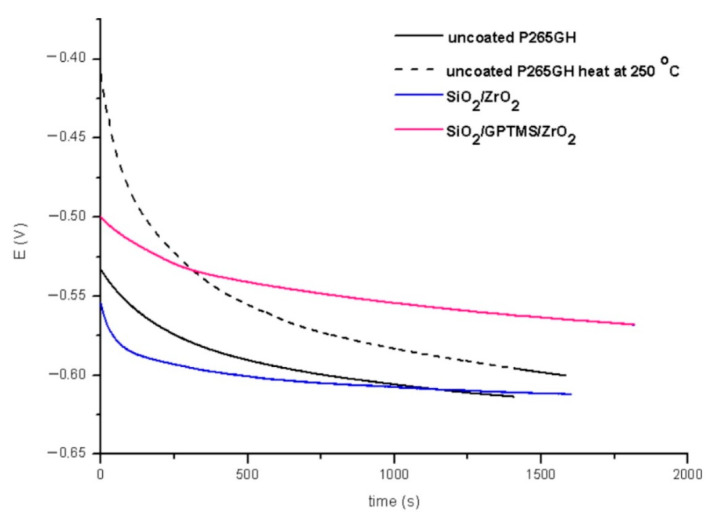
Open circuit potential for P265GH steel without coating and with oxide layers after duration in 3% NaCl 24 h.

**Figure 11:**
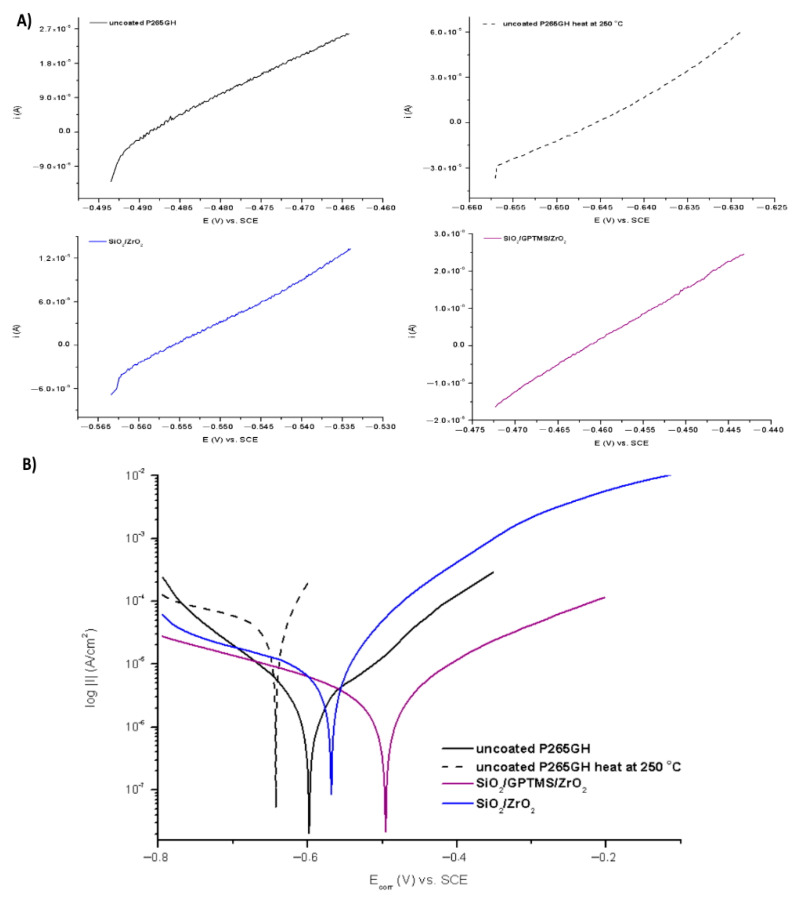
Polarization curves for P265GH steel without coating and with oxide layers, polarization resistance (**A**) and Tafel plots (**B**), after duration in 3% NaCl 0 h.

**Figure 12:**
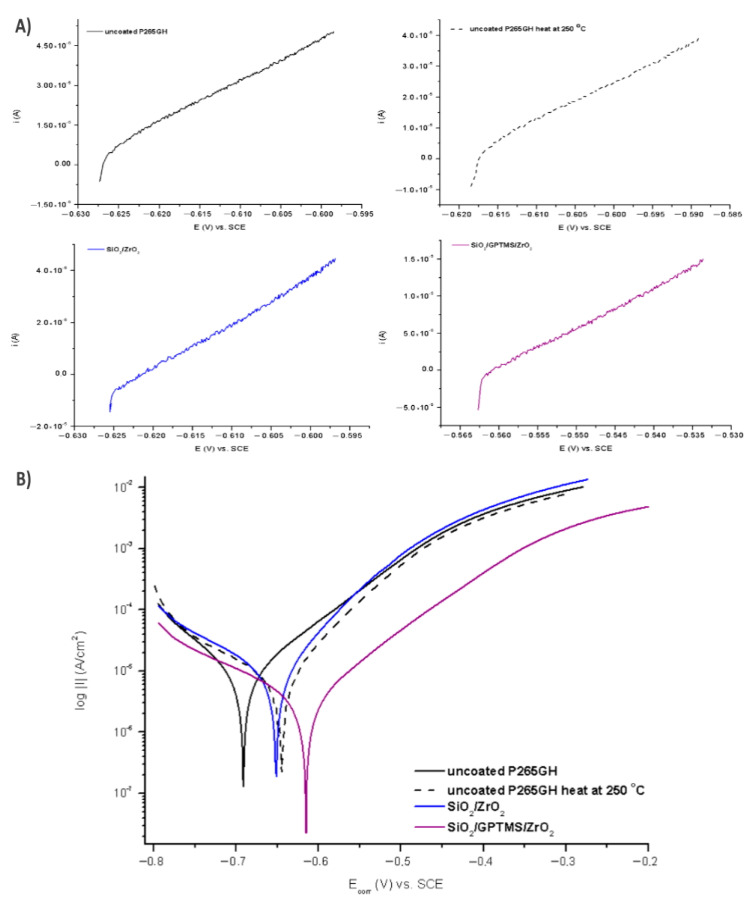
Polarization curves for P265GH steel without coating and with oxide layers, polarization resistance (**A**) and Tafel plots (**B**), after duration in 3% NaCl 24 h.

The tests were performed for the uncovered P265GH steel and 316L steel and for both steels with deposited coatings: SiO_2_/ZrO_2_ and SiO_2_/GPTMS/ZrO_2_. Figure 9 and Figure 10 present the open circuit potential for coated and uncoated P265GH after the first contact and after 24 h contact with a 3% NaCl solution, respectively. Figure 11 and Figure 12 show: (A) the obtained polarization resistance and (B) the polarization curves—Tafel plots—for the uncovered P265GH samples and the samples with oxide layers. After the first contact with a 3% NaCl solution, the shapes of the polarization curves for both the uncoated P256GH steel and the samples coated with oxide layers were very similar (Figure 11). However, it can be clearly seen that the lower densities of anode currents were obtained for the carbon steel coated with SiO_2_/GPTMS/ZrO_2_ layers. The analysis of the obtained electrochemical results, presented in Table 5, indicated that the modification of the P256GH steel substrate with SiO_2_/GPTMS/ZrO_2_ mixed coatings treated at 120 °C increased resistance to corrosion (R_p_) from 0.889 kΩ·cm^2^ to 7.273 kΩ·cm^2^ and decreased the densities of corrosive currents (i_corr_), which were in the range of 29 μA/cm^2^ to 3.6 μA/cm^2^. To confirm the barrier properties of the obtained layers, Figure 12 presents polarization curves after 24 h exposure in a 3% NaCl solution. This longer exposure confirmed the increased corrosion resistance of samples with SiO_2_/GPTMS/ZrO_2_ layers. Unfortunately, for SiO_2_/ZrO_2_, the densities of corrosive currents increased after 24 h of duration in the solution. Table 5 presents the numerical results after 24 h. The results obtained for the samples with SiO_2_/ZrO_2_ layers demonstrated that this type of coating material did not show long term corrosion protection. The results for both types of coatings after 24 h in a corrosive medium showed values similar to the R_p_ and i_corr_ of the P265GH steel (approximately 0.6 kΩ·cm^2^). The highest R_p_ (1878 kΩ·cm^2^) after 24 h of exposure in 3% NaCl was obtained for the sample with SiO_2_/GPTMS/ZrO_2_ coating. The observations of the uncoated steel before stabilization and after stabilization at 250 °C confirmed that the heat treatment affected the reduction of corrosive processes after long term exposure in 3% NaCl. The presented studies showed that P265GH steel is not a corrosion-resistant material in 3% NaCl. The highest R_p_ parameters were obtained for samples with SiO_2_/GPTMS/ZrO_2_ layers. However, the application of thin oxide films increased the properties of its anticorrosion behavior about fourfold (based on R_p,_ where anticorrosion properties for uncoated P265GH steel treated at 250 °C was 1) after 24 h exposure to 3% NaCl solutions.

**Figure 13:**
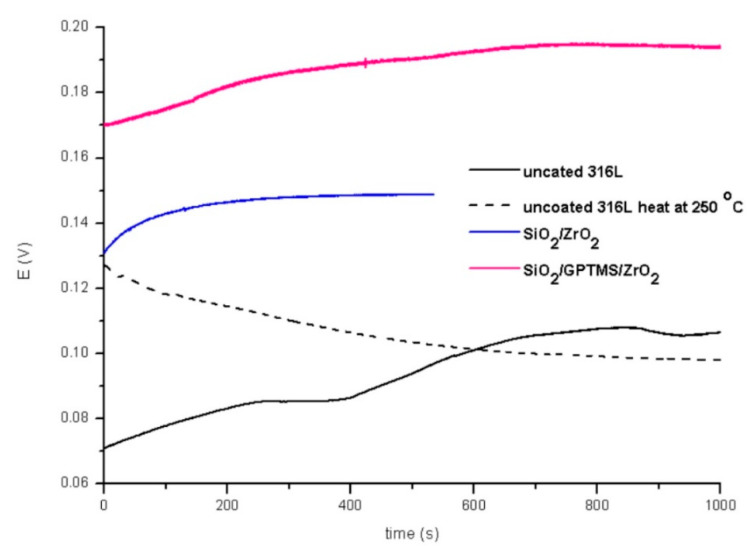
Open circuit potential for 316L steel without coating and with oxide layers after duration in 3% NaCl 0 h.

**Figure 14:**
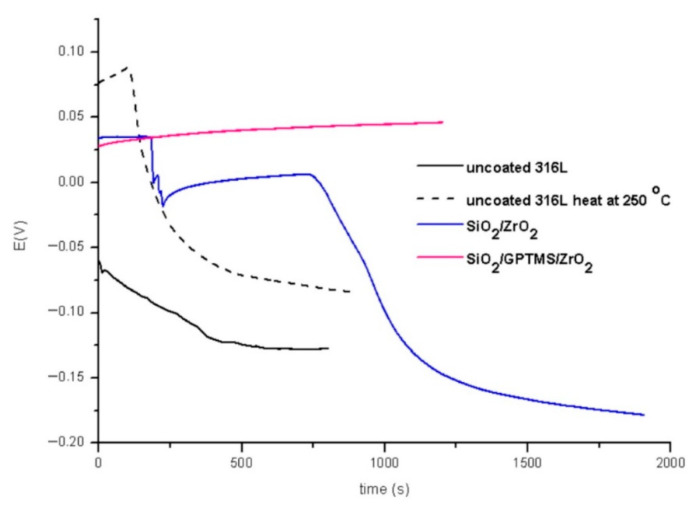
Open circuit potential for 316L steel without coating and with oxide layers after duration in 3% NaCl 24 h.

**Figure 15:**
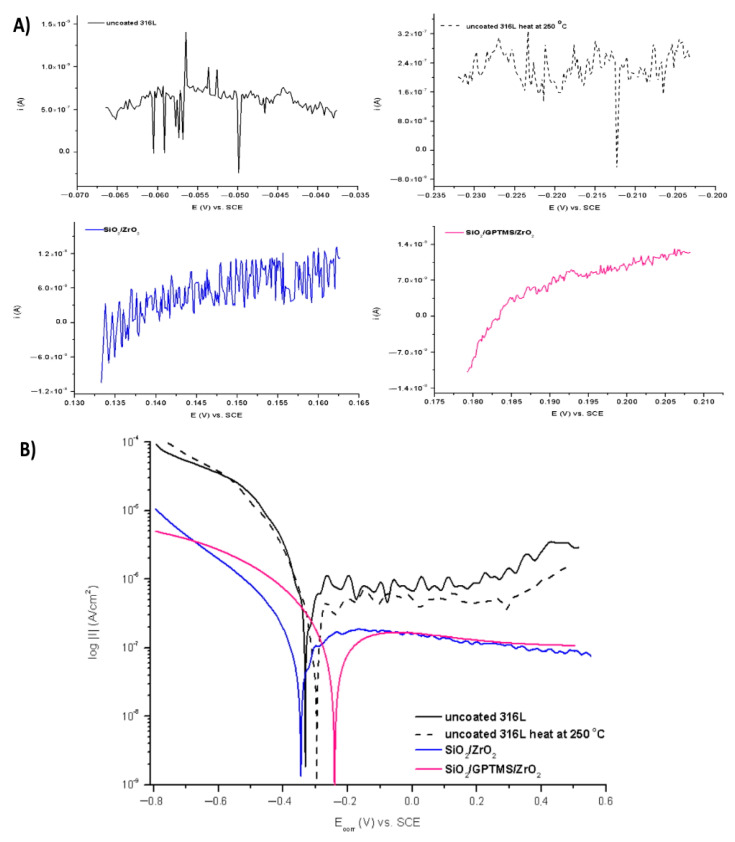
Polarization curves for 316L stainless steel without coating and with oxide layers, polarization resistance (**A**) and Tafel plots (**B**), after duration in 3% NaCl 0 h.

**Figure 16:**
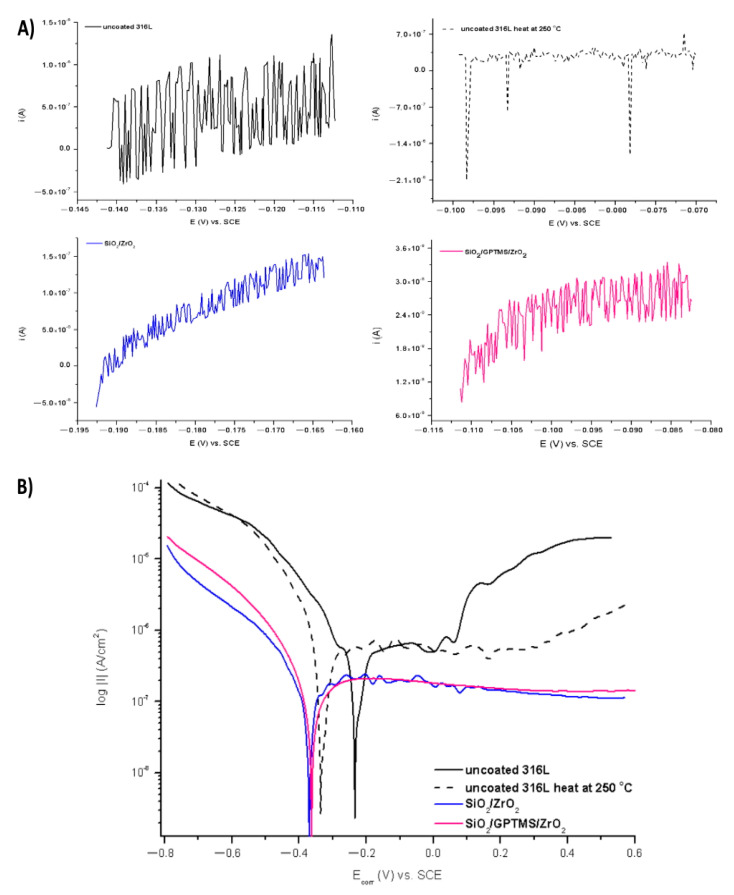
Polarization curves for 316L stainless steel without coating and with oxide layers, polarization resistance (**A**) and Tafel plots (**B**), after duration in 3% NaCl 24 h.

In the second part of the experiment, SiO_2_/ZrO_2_ and SiO_2_/GPTMS/ZrO_2_ coatings applied to 316L steel were tested. Figure 13 and Figure 14 showed the open circuit potential for 316L uncoated and with coatings after the first contact and after 24 h contact with a 3% NaCl solution, respectively. In the polarization resistance (Figure 15A) and the obtained polarization curves (Figure 15B), immediately after immersion in 3% NaCl, one could observe changes in their course. The heat treatment of the 316L steel at 250 °C caused the reduction of corrosion currents in the anode part on the Tafel plot. The lower densities of corrosion currents in both the cathodic and the anodic part were observed for SiO_2_/ZrO_2_ and mixed SiO_2_/GPTMS/ZrO_2_ layers in relation to the uncoated 316L steel. The analysis of the obtained values, presented in Table 6, allowed us to observe that the highest R_p_ was obtained for the steel with SiO_2_/ZrO_2_ layers (2.0 MΩ·cm^2^) immediately after insertion in the solution. Additionally, for the remaining coating, the value was increased in relation to the non-heated 316L steel. The densities of the corrosion currents of the uncoated substrate heated at 250 °C were about six times lower than those of 316L steel. This confirmed the significant influence of heat treatment on the increase in the corrosion properties of austenitic steel and materials with self-passivation features. The presented polarization resistance (Figure 16A) and polarization curves (Figure 16B) after 24 h of exposure to a 3% NaCl solution indicated a decrease in corrosion current density for SiO_2_/ZrO_2_ and SiO_2_/GPTMS/ZrO_2_ samples (in comparison to uncoated 316L). The SiO_2_/GPTMS/ZrO_2_ sample was also characterized by a shift towards the positive values of the cathodic–anode transition potential (E_C-A_) (Figure 16B), which was confirmed by better protective properties [57]. Table 6 presents the results obtained after 24 h of exposure to a 3% NaCl solution. The results indicated that SiO_2_/ZrO_2_ coatings did not show long term corrosion protection. Their polarization resistance decreased after 24 h of exposure in an aggressive environment, while R_p_ for the steel pre-heated at 250 °C after 24 h of exposure decreased about 20 times. The results obtained after a 24 h exposure confirmed the high protection of the SiO_2_/GPTMS/ZrO_2_ coatings. The highest polarization resistance (5.9 MΩ·cm^2^) and the lowest densities of corrosive currents (4.5 nA/cm^2^) were obtained for these layers. With reference to the uncoated and non-heated steel substrate, the polarizing resistance was about 85 times higher. The obtained results showed the high corrosion resistance of the 316L steel with SiO_2_/GPTMS/ZrO_2_ layers. The high polarization resistance and the low densities of the corrosion currents determined indicated the possible use of these layers as long term protection against aggressive environments [57,58,59,60]. A clear improvement in the corrosion resistance for both steels (P256GH and 316L) was observed after applying the proposed sol-gel coatings on them. For both steels, SiO_2_/GPTMS/ZrO_2_ layers resulted in increasing barrier properties after long term exposure to 3% NaCl. Further investigation may explain if the long term protection effect is caused by the “self-healing” process described in the literature [15,61] as the SiO_2_/ZrO_2_ coating did not show that. The obtained result seemed to be correlated with results from SEM suggesting that the deposition of SiO_2_/ZrO_2_ (Figure 3A1), especially on the P265GH steel, increased the specific surface area, which increased the number of potential interactions between corrosion agents (Cl^−^) and the surface, accelerating the damage of the material [35]. In result of long term interaction of metallic substrate with Cl^−^, the corrosion products may have grown under the coatings. According to results obtained by Croll [62], corrosion products are less dense than the metal and push the coatings away from the surface metal, which can account for the behavior of SiO_2_/ZrO_2_ coatings in NaCl solution.

## 4. Conclusions

The protective, organically modified oxide coatings were obtained by the sol-gel method. The influence of zirconia and organic additives on mechanical and electrochemical properties of silica coatings was studied.

The microscopy studies showed differences in the structure morphology between coated substrates as well as between different coatings. However, the differences were more due to the substrate than to the coating material. The EDX and the Raman spectroscopy showed rather uniform structures of both coatings. The analysis of Raman spectra showed that the coatings were built of a SiO_2_-ZrO_2_ hybrid material with organic groups corresponding to the precursors involved in the synthesis (non-hydrolyzing moieties).

The highest Vickers hardness was observed in the case of the silica-zirconia coatings with methyl moieties (SiO_2_/ZrO_2_)—it was similar to the uncoated 316L steel samples—while the SiO_2_/GPTMS/ZrO_2_ coating had five times lower hardness compared to the substrate. The cause seemed to be the organic moieties in this coating compared to the -CH_3_ moieties from SiO_2_/ZrO_2._

The SiO_2_/GPTMS/ZrO_2_ coatings showed the highest adhesion to the metallic substrate at the level of gradient layers (performed using laser oxidation).

Additionally, the hybrid SiO_2_/GPTMS/ZrO_2_ coatings exhibited the best properties in terms of corrosion mitigation in seawater. After 24 h in a 3% NaCl solution, the values of polarization resistance were nearly 84 times higher (in comparison to the uncoated 316L steel). This confirmed the positive influence of the organic part on the barrier properties of thin films using corrosion agents (such as Cl^-^). The results obtained for P265GH may have resulted from the low corrosion resistance of this kind of steel. This, in turn, was connected with a substantial rise in oxide iron scale, increasing during the corrosion process, and was also due to high volume that damaged the coatings.

For the P265GH samples, the nanoindentation results were not presented due to significant scattering of results, which probably correlated with an oxide iron scale arising on the substrate surface under the coating, influencing the inhomogeneities on the surface and enhancing difficulties in contact measurements.

However, it seems that only a combination of zirconia (ZrO_2_) with silica (SiO_2_) modified by large organic moieties (GPTMS) gives a larger chance to obtain coating materials with high adhesion to a metallic substrate and with corrosion mitigation ability. However, the influence of the substrate on the degree of degradation in corrosive environments should also be taken into account. The corrosion mitigation was observed even after extending exposure to corrosion agents, which could have resulted from the reinforcement of an organically modified silica network with zirconia.

## Figures and Tables

**Table 1:** Chemical compounds and volume ratio of substrate used to sol-gel synthesis.

Sol	Chemical Compounds
SiO_2_	TEOS + dMdEOS + EtOH + HCl (1:1:4:0.02)
SiO_2_/ZrO_2_	TEOS + dMdEOS + EtOH + HCl (1:1:4:0.02)
	B. ZrOBu + BuOH + AcAc + CH_3_COOH (1:4:0.5:0.5)
SiO_2_/GPTMS/ZrO_2_	C. GPTMS + TEOS + EtOH + HCl (1:1:4:0.02)
	B. ZrOBu + BuOH + AcAc + CH_3_COOH (1:4:0.5:0.5)

**Table 2:** Chemical compositions (%) of P265GH and 316L steel.

Element	P265GH	316L
C	0.20	0.03
Mn	1.40	2.00
Si	0.40	1.00
P	0.025	0.045
S	0.02	0.03
Cr	0.30	16.00–18.00
Mo	0.08	2.00–3.00
Ni	0.30	10.00–15.00
Al	0.02	-
Cu	0.30	-
Nb	0.01	-
Ti	0.04	-
V	0.02	-
N	-	0.11
Fe	96.89	60.79–68.79

**Table 3:** Value of characteristic destructive forces (L_c_) received by scratch test for metallic substrates with sol-gel coatings.

Coating	Value of the Characteristic Forces		
	L_C1_ (N)	L_C2_ (N)	L_C3_ (N)
**Substrate: P265GH Steel**			
**SiO_2_/ZrO_2_**	1.49 ± 0.59	4.17 ± 0.29	8.64 ± 0.81
**SiO_2_/GPTMS/ZrO_2_**	1.29 ± 0.02	6.49 ± 0.94	15.76 ± 3.22
**Substrate: 316L Steel**			
**SiO_2_/ZrO_2_**	2.58 ± 0.66	5.10 ± 0.97	7.49 ± 3.20
**SiO_2_/GPTMS/ZrO_2_**	1.92 ± 0.31	7.35 ± 0.29	12.46 ± 1.11

(L_C1_—characteristic crack of coating, L_C2_—characteristic chipping of coatings, L_C3_—penetration of the coating into the substrate in the middle of the scratch (the mean value ± standard deviation for n = 3, where n—a sum of the scratches for individual sample).

**Table 4:** Hardness and Young modulus for uncoated 316L steel and steel with coatings.

Samples	E_IT_ (GPa)	H_IT_ (Vickers)
Uncoated steel	119 ± 34	236 ± 22
SiO_2_/ZrO_2_	51 ± 27	236 ± 14
SiO_2_/GPTMS/ZrO_2_	7 ± 2	41 ± 12

**Table 5:** Corrosion test results for P265GH steel without coating and with oxide layers after O h and 24 h duration in 3% NaCl.

Material	OCP		
	E_corr_ (mV)	R_p_ (kΩ·cm^2^)	j_corr_ (μA/cm^2^)
**After 0 h Duration in 3% NaCl**			
uncoated P265GH	−596.1	0.889	29
uncoated P265GH heat at 250 °C	−646.3	0.319	82
SiO_2_/ZrO_2_	−570.2	1.706	15
SiO_2_/GPTMS/ZrO_2_	−497.4	7.273	3.6
**After 24 h Duration in 3% NaCl**			
uncoated P265GH	−692.7	0.597	44
uncoated P265GH heat at 250 °C	−643.9	0.427	61
SiO_2_/ZrO_2_	−653.3	0.576	45
SiO_2_/GPTMS/ZrO_2_	−613.2	1.878	13

**Table 6:** Corrosion test results for 316L stainless steel without coating and with oxide layers after duration to 0 h and 24 h in 3% NaCl.

Material	OCP		
	E_corr_ (mV)	R_p_ (MΩ·cm^2^)	j_corr_ (nA/cm^2^)
**After 0 h Duration 3% NaCl**			
uncoated 316L	−328.5	0.31	85
uncoated 316L heat at 250 °C	−294.9	1.4	18
SiO_2_/ZrO_2_	−344.3	2.0	13
SiO_2_/GPTMS/ZrO_2_	−239.8	1.5	17
**After 24 h Duration in 3% NaCl**			
uncoated 316L	−231.9	0.07	340
uncoated 316L heat at 250 °C	−333.4	0.08	320
SiO_2_/ZrO_2_	−372.0	0.17	150
SiO_2_/GPTMS/ZrO_2_	−363.8	5.9	4.5

## Data Availability

The data presented in this study are available on request from the corresponding author. The data are not publicly available due to privacy policy of research founder-the West Technology & Trading Poland (WTT).
